# Optimising automation of a manual enzyme-linked immunosorbent assay

**DOI:** 10.4102/ajlm.v1i1.15

**Published:** 2012-10-15

**Authors:** Corena de Beer, Monika Esser, Wolfgang Preiser

**Affiliations:** 1 Department of Pathology (Division of Medical Virology), University of Stellenbosch, South Africa; 2 Department of Pathology (Immunology Unit), University of Stellenbosch, South Africa

## Abstract

**Objective:**

Enzyme-linked immunosorbent assays (ELISAs) are widely used to quantify immunoglobulin levels induced by infection or vaccination. Compared to conventional manual assays, automated ELISA systems offer more accurate and reproducible results, faster turnaround times and cost effectiveness due to the use of multianalyte reagents.

**Design:**

The VaccZyme™ Human Anti-*Haemophilus influenzae* type B (Hib) kit (MK016) from The Binding Site Company was optimised to be used on an automated BioRad PhD^TM^ system in the Immunology Laboratory (National Health Laboratory Service) in Tygerberg, South Africa.

**Methods:**

An automated ELISA system that uses individual well incubation was compared to a manual method that uses whole-plate incubation.

**Results:**

Results were calculated from calibration curves constructed with each assay. Marked differences in calibration curves were observed for the two methods. The automated method produced lower-than-recommended optical density values and resulted in invalid calibration curves and diagnostic results. A comparison of the individual steps of the two methods showed a difference of 10 minutes per incubation cycle. All incubation steps of the automated method were subsequently increased from 30 minutes to 40 minutes. Several comparative assays were performed according to the amended protocol and all calibration curves obtained were valid. Calibrators and controls were also included as samples in different positions and orders on the plate and all results were valid.

**Conclusion:**

Proper validation is vital before converting manual ELISA assays to automated or semi-automated methods.

## Introduction

Quantitative analytical methods have advanced significantly since the development of the enzyme immunoassay (EIA) and enzyme-linked immunosorbent assay (ELISA) in 1971 by the groups of Perlmann and Engvall, and Schuurs and Van Weemen, respectively.^1-3^ Before this, the only method for performing immunoassays was the radioimmunoassay (RIA), which was first described by Yalow and Berson in 1960.^4^

However, the RIA had several shortcomings, for example the potential health threat of radioactivity, short half-lives of radioisotopes, cumbersome radioactive waste disposal, expensive counting equipment, etc.^3,5,6^ An important shift from radioisotope-labelled liquid-phase assays to solid-phase assays occurred in 1968. Miles and Hales^7^ developed an immuno-radiometric technique, which used radioactively labelled antibodies instead of labelled antigens for measuring insulin in human plasma. Plastic tubes were subsequently coated with the antigen or antibody to create a solid-phase or immunosorbent platform.^8^

Modern commercial ELISA/EIA kits use 96-well microtitre plates, where either an antigen or an antibody is noncovalently bound to a solid-phase support. These methods are widely employed by laboratories and manufacturing companies for microbiological, virological and other serological diagnostic tests, validation of assays and general quality control. Although automated pipetting devices have been used for more than two decades, the high cost associated with the technique remains a major limiting factor in developing countries and smaller laboratories.^3^

## Materials and methods

The Immunology Unit of the National Health Laboratory Service (NHLS) in Tygerberg, South Africa, uses ELISAs for serological quantification of antibody levels. Before installation of the BioRad PhD™ system, all assays had been performed manually. The system performs sample dilution, dispenses patient samples and reagents into the microplate, and performs temperature-specific incubation and washing according to pre-defined protocols.

### Automation of the MK016 enzyme-linked immunosorbent assay

The 96-well microtitre plate included in the VaccZyme™ Human Anti-*Haemophilus influenzae* type B (Hib) kit (MK016) from The Binding Site Company (Birmingham, United Kingdom) is precoated with the Hib capsular polysaccharide antigen conjugated to human serum albumin. In addition to controls and other reagents, the kit also contains five calibrators (0.1 mg/L – 9.0 mg/L) to construct a five-point calibration curve. Concentration (logarithmic scale) is plotted against optical density (linear scale) to produce the calibration curve. The quantification range for anti-Hib antibody concentration is 0.11 mg/L – 9.0 mg/L.^9^

Prediluted samples and controls were pipetted into the plate and incubated for 30 minutes. Unbound proteins were removed by a wash step before addition of conjugate (purified peroxidase-labelled rabbit anti-human *γ*-chain-specific immunoglobulin G). After a further 30 minutes of incubation, another wash step was applied to remove all excess conjugate. Substrate (3.3’,5.5’ tetramethylbenzidine) was then added, which induced a colour change (from the characteristic serum colour to blue) over 30 minutes. Phosphoric acid was then added to stop colour development. The optical density (OD) was measured spectrophotometrically at 450 nm and the intensity of the final colour is proportional to the concentration of antibody present in the sample.

A list of assays that are validated on the PhD^TM^ system is available from BioRad (www.bio-rad.com; PhD™ Validated Assay List).

## Results

**FIGURE 1:**
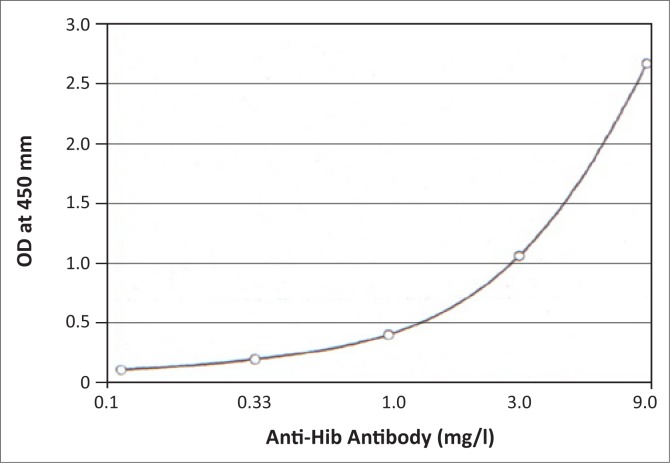
VaccZyme™ Anti-Hib Enzyme-linked immunosorbent assays calibration curve as provided on the quality control sheet.^9^

Analyses with ELISA kits from the BioRad list produced valid results in our laboratory and these methods were automated without any problems. However, when performing the non-validated MK016 assay on the PhD^TM^ system, the calibrators did not produce the required OD values recommended by the quality control sheet included in the kit (Figure 1).

The highest calibrator reached an OD of only 1.417 ± 0.245 (range 1.252–1.813) instead of 2.500 as specified on the quality control certificate. Although the lower calibrators were associated with smaller margins of error, they showed a similar trend. All results calculated from this calibration curve were therefore too low and hence invalid.

Comparison of the individual steps of the automated and manual methods identified a difference of 10 minutes in all incubation periods. The PhD™ system times incubation for each well individually and proceeds to the next step only once the exact incubation time has been reached for that specific well. However, timing of manual assays usually starts only after reagents have been added to the last well of the plate; i.e. well incubation is timed rather than plate incubation.

In an effort to address this difference, all incubation steps were increased from 30 minutes to 40 minutes on the PhD™ system. The duration of the washing steps of the two methods was very similar and therefore not regarded as contributing to the discrepant results. The duration of the washing steps was therefore left unchanged.

**FIGURE 2:**
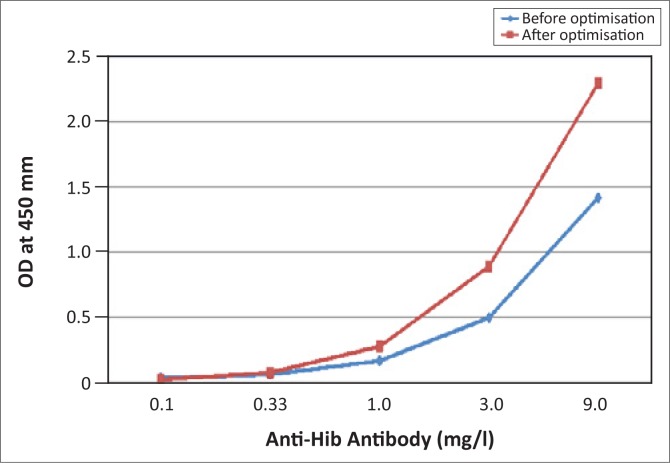
VaccZyme™ Anti-Hib enzyme-linked immunosorbent assay calibration curves before and after optimisation.

A total of 16 calibration curves were subsequently generated from the PhD™ system to validate the adjusted protocol. All the OD values obtained produced acceptable calibration curves (Figure 2) and the mean OD value for the highest calibrator reached 2.295 ± 0.171 (median = 2.405; range = 1.934–2.553). The recommended values for the high and low controls are 2.4 mg/L – 3.6 mg/L and < 0.35 mg/L, respectively. The high and low controls as used on the PhD™ system produced results of 2.538 ± 0.094 mg/L and < 0.35 mg/L, respectively.

To confirm our findings, calibrators and controls were included in different positions and orders on the plate. Values obtained for these tests were within 5% of the required ranges, which suggested that the amended protocol had a uniform effect on all individual wells of the plate (data not shown).

## Discussion

Automated systems, such as the BioRad PhD™ instrument, are extremely useful and accurate in assessing immune responses to specific antigens following disease or vaccination. The major advantages of automation or semi-automation include the use of volumes as low as 1 μL, increased accuracy and reproducibility of results, better use of expensive skilled labour, faster overall laboratory turnaround time, ability to perform multiple assays simultaneously and cost effectiveness due to use of multianalyte reagents. Use of an automated system also eliminates pipette volume variation and handling errors.

Accurate, reproducible and reliable laboratory results are crucial for patient management and care, such as initiating treatment in patients with possible immunodeficiency and revaccination of children with insufficient protection following routine childhood vaccination. It is furthermore important for evaluation of study cohort results or establishing reference ranges for specific populations or age groups.

This study emphasises the importance of optimisation and validation whenever changing protocols or reagents in order to produce valid and accurate results. In the case of the MK016 assay it was not possible to transfer the protocol established for the manual method to the automated system without modification. After troubleshooting all the individual steps of both methods, a methodological difference was identified and addressed. The modified protocol was scrutinised by repeated measurements of samples of known concentration in different assays and plate positions before amending the existing protocol.
